# The auditory‐verbal hallucinations of Welsh–English bilingual people

**DOI:** 10.1111/papt.12234

**Published:** 2019-06-11

**Authors:** Lowri M. Hadden, Ben Alderson‐Day, Mike Jackson, Charles Fernyhough, Richard P. Bentall

**Affiliations:** ^1^ School of Psychology, Cardiff University UK; ^2^ School of Psychology Bangor University UK; ^3^ Department of Psychology Durham University UK; ^4^ Department of Psychology University of Sheffield UK

**Keywords:** auditory hallucinations, bilingualism, hearing voices, psychosis

## Abstract

**Objectives:**

Psychological models of voice‐hearing propose that auditory‐verbal hallucinations occur when inner speech is attributed to a source external to the self. Approximately half of the world's population is multilingual, and the extent to which they use a second language for inner speech depends on their experience and competency in it. Bilingualism therefore provides a natural window into the processes operating in auditory‐verbal hallucinations, but no systematic study of voice‐hearing in bilinguals has hitherto been conducted.

**Design:**

A mixed‐methods observational study of psychiatric service users who hear voices and who are Welsh–English bilingual.

**Methods:**

Thirty‐seven participants were interviewed about their history and use of Welsh and English and divided into three groups: those who learnt Welsh first (L1 Welsh), those who learnt English first (L1 English), and those who learnt the two languages simultaneously. Detailed phenomenological data were collected using The Mental Health Research Institute Unusual Perceptions Schedule.

**Results:**

Both qualitative and quantitative data indicated very considerable variation in the extent to which voices were in Welsh, English, or both, with some voice‐hearers reporting that the predominant language of their voices had changed with time. There were modest but statistically significant associations between the predominant language of voices and age of language acquisition (late Welsh learners did not hear voices in Welsh), frequency of language use (more frequent use of Welsh was associated with more Welsh voices), and subjective language proficiency (proficiency in English was associated with a tendency to hear English voices).

**Conclusions:**

Although this was a small study, it was the first of its kind. There is a need for more research on the implications of bilingualism for psychosis in particular and mental illness more generally. The results are broadly consistent with the hypothesis that hallucinated voices are misattributed inner speech.

**Practitioner points:**

- Assessments of people with mental health difficulties should routinely inquire whether they are multilingual and, if so, which language they prefer to use.
- People with mental health difficulties may have difficulty expressing complex issues and emotions in a second language, despite apparent fluency.
- When working with bilingual people who hear voices, mental health professionals should consider the language used by the voices when conducting assessments and proposing formulations.

## Background

Auditory hallucinations – hearing sounds in the absence of appropriate external stimuli – are a common experience in patients with psychosis and are typically verbal in nature. What ‘voices’ say, how they say it, and who is perceived to be speaking are core aspects of their phenomenology (Nayani & David, 1996; Woods, Jones, Alderson‐Day, Callard, & Fernyhough, 2015) and often the focus of psychotherapeutic approaches to their management (e.g., Hayward, Berry, & Ashton, 2011; Leff, Williams, Huckvale, Arbuthnot, & Leff, 2014).

Psychological and neuroscientific accounts of voice‐hearing have focused on internal monitoring of speech and language processes (Ditman & Kuperberg, 2005; Laroi & Woodward, 2007). Specifically, it has been argued that voices may result from the misattribution of inner speech (i.e., silent self‐talk, verbal thought) to an external source (e.g., Allen, Aleman, & McGuire, 2007; Bentall, 1990; Frith, 1992). It has sometimes been argued that these models have struggled to account for the wide variety of experiences reported by voice‐hearers (McCarthy‐Jones, Krueger, Larøi, Broome, & Fernyhough, 2013) and that they pay insufficient attention to important social and experiential facets of voice‐hearing such as their relation to culture (Laroi *et al*., 2014). However, one factor that may account for some of the heterogeneity in voice‐hearing is language history, that is, an individual's experience and use of language throughout their lifespan. If voices result from the misattribution of inner speech, then the characteristics of an individual's inner speech should be reflected in characteristics of the voices. This may be difficult to demonstrate when only one language has been used since birth, but, in cases of bilingualism, variations in experience and competence in more than one language create a natural experiment: Do voice‐hearers have experiences that reflect variations in their language experience?

The amount of exposure that bilinguals have to each language impacts on their proficiency, with greater proficiency typically in the language used in the home environment (Gathercole & Thomas, 2009). Importantly, the more proficient they are in a language, and the longer they have been using it to communicate in their everyday lives, the more likely they are to use it for inner speech (de Guerrero, 2004; Larsen, Schrauf, Fromholt, & Rubin, 2002). Hence, if auditory‐verbal hallucinations are inner speech that has been misattributed to an external source, it follows that the language in which voices are heard should be similarly affected by experience and proficiency in the different languages.

More subtle effects of bilingualism are possible. Because language structures thinking and has profound effects on perception and cognition – influencing such basic processes as the way that people experience colour (Thierry, Athanasopoulos, Wiggett, Dering, & Kuipers, 2009) and think about space and time (Boroditsky & Gaby, 2010) and human agency (Fausey, Long, Inamori, & Boroditsky, 2010) – each language spoken by a bilingual individual independently modulates the way that he or she experiences the world (Grosjean, 2001). For example, bilinguals’ views of themselves and the way that they express their personalities can differ according to whether they are conversing in their first (L1) or second language (L2) (Pavlenko; Ramírez‐Esparza, Gosling, Benet‐Martínez, Potter, & Pennebaker, 2006). Decision‐making (Keysar, Hayakawa, & An, 2012), emotional experience (Dewaele, 2010; Wu & Thierry, 2012), beliefs (Ellis *et al*., 2015), and implicit cognitive biases (Wu & Thierry, 2012) are all differentially influenced by L1 and L2.

A few case studies have suggested that bilinguals who have received a diagnosis of schizophrenia show variation in their symptom expression across their languages. For example, De Zulueta, Gene‐Cos, and Grachev (2001) reported that a bilingual woman only experienced voices in her first language, Italian, but not in English. Wang, Morales, and Hsu (1998) reported a case series of six bilingual patients who were migrants to the United States, of which two heard voices in both languages, two heard voices mainly in L1 but occasionally in L2, one heard voices exclusively in L1, and one heard voices exclusively in L2. However, to our knowledge, there have been no systematic studies of the relationship between hearing voices and bilingualism and specifically whether voices are experienced in L1 and/or L2.

In the present study, we approached this question by investigating auditory‐verbal hallucinations in a sample of Welsh–English bilingual voice‐hearers. Based on the observation that relative language proficiency in bilinguals predicts their use of their languages during inner speech, we expected that the extent to which voices are experienced in the two languages would be predicted by relative language use and proficiency. Our approach included a qualitative analysis of the phenomenology of our participants’ voices, focusing specifically on their language, followed by a quantitative analysis of the relationship between these characteristics and age of acquisition and proficiency in the two languages.

The contiguous areas of Gwynedd and Anglesey, which are located in the north‐west of Wales, have a particularly high density of bilingual Welsh–English speakers (85.6% and ~74%, respectively; Office of National Statistics, 2013). Hence, this region offered a unique opportunity to draw from a population of highly proficient bilinguals, from similar non‐migrant and socio‐economic backgrounds.

## Methods

### Participants

Thirty‐seven Welsh–English bilingual psychiatric service users who heard auditory‐verbal hallucinations were recruited. They were recruited from community mental health teams (CMHTs) throughout Wales (28), mental health charities (6), or had self‐referred (3) by responding to posters and adverts placed in local services. Thirty‐one had received diagnoses of schizophrenia‐spectrum disorders such as schizophrenia (8), schizoaffective disorder (3), psychosis (12), or psychosis with other disorders (8) from their responsible clinicians. The remaining participants had received diagnoses of bipolar disorder (3), major depression (1), and borderline personality disorder (2). A similar number of males (*N* = 20) and females (*N* = 17) were recruited and ages ranged from 18 to 68 years (*M *=* *38.03; *SD *=* *13.50).

Participants reported an age of voice onset, assessed through the MUPS (see below), ranging from one to 40 years of age (*M *=* *20.23; *SD *=* *10.18) (clearly some of these reports are implausibly low). The reported duration of the voices ranged from 1 year to 49 years (*M *=* *17.80; *SD *=* *11.82). All participants provided informed consent, were given the opportunity to withdraw from the study at any time without giving a reason, and were debriefed at the end. The study was approved by an NHS ethics committee and by the relevant health service research governance committees.

### Measures

*The Language Background Questionnaire* (Thomas & Gathercole, 2007) is a self‐report measure of language proficiency and use. Questions determine age of language acquisition, perceived language ability (reading, writing, speaking, and understanding), and use of language in everyday life. We used this questionnaire to determine (1) age of language acquisition, (2) self‐reported proficiency, and (3) frequency of language usage. As a consequence of our convenience sampling method of recruiting voice‐hearers into the study, there were an uneven number of participants falling into age of acquisition groupings. The majority of participants were simultaneous bilinguals, having learned both Welsh and English from birth (*N* = 22). L1 Welsh bilinguals (*N* = 10) were classed as those who had learned Welsh from birth and begun to learn English later (between the ages of 4 and 8 years). The L1 English group (*N* = 5) included people who had learned English from birth and begun to learn Welsh later (between the ages of 8 and 14 years).

We calculated an overall subjective language *proficiency score* for both Welsh and English separately. Composite scores were computed as the average proficiency scores (1 = low proficiency to 5 = high proficiency) across dimensions of speaking, understanding, reading, and writing for both Welsh and English separately. Across the sample, there was greater proficiency in English (*M *=* *4.86; *SE *=* *.06) compared to Welsh (*M *=* *4.65, *SE *=* *.08), *t*(36) = −2.46, *p = *.019.

Based on the answers from the LBQ, we calculated a *frequency of language use* variable to determine the proportion of each language participants used throughout their lives. One section of the LBQ estimates the proportion of each language a person has used from childhood to adulthood with parents, siblings, teachers, and friends, for example, ‘What language would you speak with your mother in the home at the following ages (before school, primary school, secondary school, and as an adult)?’. The response options were Welsh, Mostly Welsh, Bilingual, Mostly English, and English. We collapsed the Welsh and Mostly Welsh into one category (Welsh) and English and Mostly English into one category (English). In each domain, a score of + 1 was awarded for reported Welsh language use; a score of 0 was given to equal language use (bilingual); and a score of −1 was given for English language use. These scores were then averaged across domains. Therefore, a more positive score indicates greater frequency of Welsh language, and a more negative score indicates greater frequency of English language usage.

*The Mental Health Research Institute Unusual Perceptions Schedule* (MUPS; Carter, Mackinnon, Howard, Zeegers, & Copolov, 1995) is a semi‐structured, phenomenological interview designed to be more flexible than standard, structured clinical interviews by providing sufficient freedom for interviewees to expand upon their voice‐hearing experiences. The MUPS is divided into several subsections ranging from the physical to the personal characteristics of voices, as well as cognitive processes, and perceptions of voices. The schedule consists of 365 questions. Reliability has been reported to be high between raters on separate sections of the MUPS (*M*s* *=* *0.81–0.98; see Carter *et al*., 1995). From the MUPS interviews, we extracted the language the voice/voices used to speak to the voice‐hearers.

The interview data were initially scored as a categorical variable for language of voices. For example, for the question, ‘In what language did your voice speak to you?’, voice‐hearers would typically report the language in which they experienced their voices. We subsequently asked the voice‐hearers to provide an estimate of how often they experienced their voices in each language, and categorized them into Welsh, Mostly Welsh, Bilingual, Mostly English, or English, based on a similar criteria to the LBQ.

We transformed these categories into a continuous variable called the *voice language index* to conduct further regression analyses alongside other continuous variables. For example, if a person heard a single voice only in English, they would get a score of −1; if they heard a bilingual voice, they would get a score of 0; and if they experienced a single Welsh voice, they would receive a score of +1. If a person had more than one voice, the language of all voices was assessed and the resulting language index score would be the amount of Welsh, English, or both that the voices spoke divided by the number of voices heard. For example, one participant experienced three voices. One of these voices spoke Welsh 100% of the time (+1), another spoke English 100% of the time (−1), and the final voice was bilingual (0) and alternated between English and Welsh depending on context; the resulting *voice language index* score was 0.

*The Psychosis Screening Questionnaire* (PSQ; Bebbington & Nayani, 1995) was used to verify the presence of psychosis‐like experiences on entry to the study. The PSQ is validated in terms of its specificity and sensitivity to the presence of psychosis‐like experiences, which are separated into five types consisting of *Thought Interference*,* Persecution*,* Perceptual Abnormalities, Strange Experiences,* and *Hallucinations*. Each section consists of an initial screening question, for example, ‘Over the past year, have you felt your thoughts were being directly interfered with or controlled by another person?’. If the initial screening question was answered affirmatively, participants would answer the follow‐on questions.

### Procedure

Voice‐hearers were tested in a quiet room at local CMHTs across Wales, in the university, at third‐sector organizations, or in their homes. The MUPS was administered to the voice‐hearers after five minutes of rapport‐building, with an opportunity to ask questions and discuss concerns. Questionnaires were presented in either Welsh, bilingually, or in English, based upon the participant's preference. The majority of voice‐hearers chose to complete the MUPS in Welsh or bilingually. Participants were paid £20 for their participation and costs for reasonable travel expenses.

## Results

### Phenomenology

**Figure 1:**
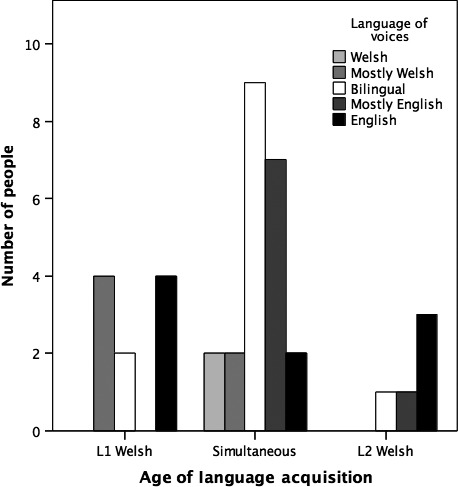
Age of acquisition and language of voices.

Two participants reported voices exclusively in Welsh, 6 mostly in Welsh, 12 in both languages, 8 mostly in English, and 9 exclusively in English (see Figure 1). Typically, participants had more than one voice. Some voices were indistinct or were perceived as belonging to a gang of voices with a ringleader. Most did, however, identify a most important voice or voices, which was typically dominant or oppressive. Voice‐hearers had different relationships with the voices, with some experienced as reassuring, and others as commanding and unkind, and some as a mixture of both.

Several participants reported that their relationship with their voices, and the extent to which they were expressed in the two languages, had changed over time. For example, one participant reported that:

P1 ‘The beginning of it was Welsh when I was younger, but when I was about 16 they changed to English. It was a lot more oppressive, negative, emotional’.

Another reported a striking example of a voice at first appearing to be monolingual English and then later developing apparent competency in Welsh. Note, also, that in this example, the voice's use of the two languages is perceived as strategic:

P2 ‘Mae na un oedd yn siarad drwy'r adeg so oeddwn i'm yn gallu meddwl ‘sti, ond os oeddwn i'n meddwl yn y Gymraeg doedd hi ddim yn deall fi so oedd hi'n mynd yn ddistaw. A wedyn on i'n meddwl on i'n iawn, yn meddwl yn y Gymraeg drwy'r adeg a wedyn ddaru hi ddechrau canu cân yn Gymraeg drosodd a drosodd so oedd o fatha yn Gymraeg ond oedd o'n fatha spiteful ofnadwy. Ond mae'r Saesneg yn fwy, mae o bob dim o un yn ‘go and re‐enact the columbine massacre at the primary school ‘lly, a mae'r lleill sydd yn jest, dw i'm yn gwybod fatha siarad am y tywydd a pethau fel yna, so mae na lot fwy o variation yn y rhai Saesneg’. [Trans: ‘There is one [voice] that speaks all the time so I wasn't able to think, but if I thought in Welsh she wasn't able to understand me and would go quiet. And then I thought I was OK thinking in Welsh all the time, and then she started singing a Welsh song over and over again, so it was like in Welsh it was very spiteful. But English is more, everything is like ‘go and re‐enact the Columbine massacre at the primary school’, and the others are just speaking about the weather and things like that, so there's a lot of variation in the English ones’]

For those participants who experienced voices in both languages, the identity of the voice often seemed to be bound to the language it used. For example:

P3 ‘Dw i wedi cael lleisiau yn Saesneg sydd yn advisory, a commentary kind of. Dw i wedi cael rhai Cymraeg sydd yn bobl dw i'n nabod. A pobl sydd wedi pasio‐ sydd wedi marw. Pobl dw i wedi dod ar draws yn fy mywyd’. [Trans. ‘I have had voices in English that are advisory, a commentary kind of. I have had some Welsh that are people that I know. And people that have passed‐ who have died. People whom I have come across in my life’.]

Similarly:

P4 ‘Mae'r lleisiau Cymraeg mae nhw'n bobl on i'n nabod. Pobl hyd yn oed sydd wedi marw blynyddoedd yn ôl. A fatha yn siarad efo fi de. Ffrindiau hyd yn oed. Dw i'n gwybod mae o'n swnio'n wirion fatha ond yn Gymraeg mae o'n teimlo'n fwy cyfforddus bron pan dw i'n siarad Cymraeg ‘de’. [Trans. ‘The Welsh voices are people I knew. People who have even died years ago. And speak to me. Even friends. I know it sounds silly, but in Welsh it feels more comfortable when I speak Welsh’.]

These quotations also illustrate the different roles taken on by the voices in the two languages, as also noted by other participants:

P5 ‘Mae'r lleisiau Saesneg yn dweud wrthyf fi wneud pethau na ddylswn i neud’. [Trans. ‘The English voices tell me to do things that I shouldn't do’]

And:

P6 ‘Mae o'n dibynnu be dw i'n gweld a be dw i'n clywed. Mae bob dim drwg rhanfwyaf yn Saesneg – y lleisiau drwg. Mae'r pethau da‐ mae ‘na rhai pethau da yn Saesneg a Cymraeg ‘sti’. [Trans: ‘It depends what I see and hear. Everything bad mainly is in English ‐ the bad voices. The good things ‐ there are some good things in English and Welsh’.]

For another participant, it was her English‐speaking voice that was most benevolent, causing her to find the Welsh language irritating because it was the medium used by persecutory voices:

P7 ‘Dw i'n gwylltio lot efo'r iaith Gymraeg coz Cymraeg on i'n neu dw i wedi clywed y lleisiau brwnt ‘ma, so os dw i'n teimlo fel dw i wedi pwdu siarad Cymraeg neith yr guardian angel siarad yn Saesneg’. [Trans. ‘I can get very annoyed with the Welsh language coz Welsh was or is the language I hear these mean voices, so if I feel like sulking with the Welsh language the guardian angel will speak in English’.]

### Age of acquisition and language of voices

Strikingly, all five participants in the L1 English group had voices that were exclusively or predominantly in English (see Figure 1), whereas the patients in the other two groups had voices that were exclusively Welsh, exclusively English, or in both languages. We ran a chi‐square analysis to determine whether the two categorical variables, age of acquisition and language of voice/s, were related. Due to violation of the assumption of expected cell counts of at least 5 in 80% of the cells, we report the maximum likelihood ratio chi‐square test (McHugh, 2013). We found a significant association between age of language acquisition and language of voices, χ^2^ (8, *N* = 37) = 18.98, *p *=* *.015, indicating that voice‐hearers who learned Welsh first or who were simultaneous bilinguals were more likely to hear Welsh or bilingual voices, whereas L1 English speakers were more likely to hear English voices

Confirming this finding, the difference between the groups on the voice language index (which, it will be recalled, is coded so that a greater proportion of English voices generates a more negative value) was significant, *F*(2, 34) = 2.98, *p* < .05, accounted for by the higher scores of the L1 Welsh relative to the L1 English participants, Tukey *p* < .05 (this should be interpreted with caution given the numbers in the groups).

### Use of the two languages

A regression model predicting the voice language index from how often participants used each language (frequency of language use) was significant, *R* ^2^ = .121, *F*(1, 35) = 4.80, *p *=* *.035, standardized β = .35, *t *=* *2.19, *p *=* *.035; those who used Welsh more frequently tended to have more Welsh voices, and those who used English more frequently tended to have more English voices.

### Subjective proficiency

We ran a multiple regression to determine whether subjective Welsh proficiency and subjective English proficiency separately predicted the language of voices. The model was significant, *F*(2, 35) = 3.41, *p* < .05, and was accounted for by effect of subjective English proficiency, standardized β = −.318, *p* < .05, but not the effect of subjective Welsh proficiency, standardized β = .27, *p* = .09.

## Discussion

Despite our modest sample size, which was a consequence of the very specific criteria required for participants to enter the study, we believe that this is the largest investigation of bilingual voice‐hearers so far reported and is the first to examine empirically how linguistic factors and the language environment impact upon their hallucinatory experiences.

Our participants had highly varied experiences, with some reporting reassuring voices, some reporting commanding voices, and some reporting a mixture of kind and mocking voices, consistent with previous reports on the phenomenology of voice‐hearing (e.g., Nayani & David, 1996; Woods *et al*., 2015). We observed that the bilinguals in this study had evolving relationships with their voices, regardless of whether those voices spoke exclusively in one language or both. These changes possibly reflected developmental factors, changes in environment and context, or both. For example, one voice‐hearer reported hearing her voices exclusively in Welsh throughout her childhood to her mid‐teens when the voices switched to English, and became more oppressive and unkind, possibly reflecting changes in exposure to the two languages and language use.

Themes of dominance and subordination are known to be important in the perception of hallucinated voices, with distress most likely to be experienced when voice‐hearers feel socially powerless and when voices, like individuals in the hearer's surrounding social universe, are experienced as dominating (Birchwood, Meaden, Trower, Gilbert, & Plaistow, 2000; Birchwood *et al*., 2004). Hence, when language use reflects culturally determined power dynamics, another possible explanation for linguistic changes could be shifting subordinate–dominant relationships between voice‐hearers and their voices. Globally, languages have often been a focus of political ideology and marker of identity (Woolard, 1994), and this has certainly been true during the history of Wales (Ford, 2016), which, since the beginning of the industrial revolution, has been economically disadvantaged compared to the rest of the United Kingdom. The recommendations of a Royal Commission on Welsh Education in 1847 resulted in the suppression of the Welsh language in schools during the late nineteenth and early twentieth century, creating a sense of dominance by English culture. This dominance, in turn, provoked the emergence of a Welsh nationalist movement that culminated with the establishment of the Welsh Assembly Government in 1997. Even at the present time, there is considerable local variation both in the use of Welsh and in the strength of Welsh identity within the North Wales area (Williams, 2009). Hence, at the psychological level it is entirely plausible that the Welsh and English languages carry associations related to subordination–dominance.

The dynamic nature of voice‐hearing, as revealed in our qualitative data, places an important constraint on the interpretation of our quantitative data, which provides a ‘snapshot’ of bilingual voice‐hearing at one point in time. The overwhelming picture was again diversity in the experience of voices, with some participants experiencing their voices in English, some in Welsh, but the majority experiencing voices in both languages. This observation is consistent with the only previous case series of bilingual voice‐hearers reported by Wang *et al*. (1998).

Nonetheless, and consistent with inner speech models of auditory‐verbal hallucinations (e.g., Allen *et al*., 2007; Bentall, 1990; Frith, 1992), there was a relationship between language acquisition and the language of the voices heard, with participants who had learned Welsh later rarely experiencing Welsh voices. Subjective proficiency in English (but not Welsh) was also associated with English voices, as was the tendency to use English in everyday life. However, these effects were weak.

The striking absence of Welsh voices in those who learnt Welsh later in childhood possibly suggests that those who, at this age, acquire a minority language (which, despite being an official language equal in status to English, is not used extensively in the media or the public space) do not tend to use it during inner speech (the opposite effect would not be expected for those who learn English late, given that English is the dominant language in the public sphere, including in education). However, this hypothesis remains conjecture at this stage and requires further investigation.

There are a number of limitations of this study that are important to note. First, because of the very specific characteristics we sought in our participants, the sample size was small, limiting our power to detect effects. Our groups were also unevenly balanced in terms of age of acquisition. The majority of voice‐hearers had learned Welsh and English from birth, with very few participants having learned English first, again limiting our power to detect effects. All participants had a high degree of competency in both languages, which again might limit our ability to detect effects. Our measure of subjective language proficiency might therefore be better considered to be a measure of confidence in the use of each language. In future studies, it will be useful to include objective measures of language proficiency.

Approximately half of the world's population is bilingual (French & Jacquet, 2004), and this neglected human attribute may have important implications for psychotherapeutic practice (Aragno & Schlachet, 1996). L1 is often associated with richer emotional experience (Wu & Thierry, 2012) and, therefore, may be the means by which clinicians can best assess patients and understand their difficulties. However, because L2 is associated with reduced emotionality, speaking in this language may ease discussion of sensitive or traumatic life events (Tehrani & Vaughn, 2009). Most likely, there are further implications that we have not considered here and, hence, there is an unrecognized need for a programme of research into the relationship between language use, risk of mental illness, and therapeutic response. We hope that, as well as providing a window into the psychological and linguistic mechanisms involved in hearing voices, this study will stimulate this kind of research.
